# Application of the PANELVIEW instrument to evaluate the guideline development process of the German polytrauma guideline

**DOI:** 10.1007/s00068-024-02470-6

**Published:** 2024-02-21

**Authors:** Käthe Goossen, Dan Bieler, Alina Weise, Monika Nothacker, Sascha Flohé, Dawid Pieper

**Affiliations:** 1 Institute for Research in Operative Medicine (IFOM), Faculty of Health, School of Medicine, Witten/Herdecke University, Ostmerheimer Str. 200, 51109 Cologne, Germany; 2 Department of Orthopaedics and Trauma Surgery, Reconstructive Surgery, Hand Surgery, Plastic Surgery and Burn Medicine, German Armed Forces Central Hospital Koblenz, Koblenz, Germany; 3 Department of Orthopaedics and Trauma Surgery, Medical Faculty and University Hospital Düsseldorf, Heinrich-Heine-University, Düsseldorf, Germany; 4 Institute for Medical Knowledge Management, Association of the Scientific Medical Societies (AWMF), c/o Philipps Universität Marburg/AWMF Berlin, Marburg, Germany; 5 Department of Trauma Surgery, Orthopaedics and Hand Surgery, Städtisches Klinikum Solingen, Solingen, Germany; 6 Faculty of Health Sciences Brandenburg, Brandenburg Medical School (Theodor Fontane), Institute for Health Services and Health System Research, Rüdersdorf, Germany; 7 Center for Health Services Research, Brandenburg Medical School (Theodor Fontane), Rüdersdorf, Germany

**Keywords:** Clinical practice guidelines, Guideline development, Methodology, PANELVIEW, Polytrauma

## Abstract

**Background:**

PANELVIEW is an instrument for evaluating the appropriateness of the process, methods, and outcome of guideline development and the satisfaction of the guideline group with these steps.

**Objective:**

To evaluate the guideline development process of the German guideline on the treatment of patients with severe/multiple injuries (‘German polytrauma guideline’) from the perspective of the guideline group, and to identify areas where this process may be improved in the future.

**Methods:**

We administered PANELVIEW to the participants of the 2022 update of the German polytrauma guideline. All guideline group members, including delegates of participating medical societies, steering group members, authors of guideline chapters, the chair, and methodological lead, were invited to participate. Responses were analysed using descriptive statistics. Comments received were categorised by domains/items of the tool.

**Results:**

After the first, second, and last consensus conference, the guideline group was invited via email to participate in a web-based survey. Response rates were 36% (*n*/*N* = 13/36), 40% (12/30), and 37% (20/54), respectively. The mean scores for items ranged between 5.1 and 6.9 on a scale from 1 (fully disagree) to 7 (fully agree). Items with mean scores below 6.0 were related to (1) administration, (2) consideration of patients’ views, perspectives, values, and preferences, and (3) the discussion of research gaps and needs for future research.

**Conclusion:**

The PANELVIEW tool showed that the guideline group was satisfied with most aspects of the guideline development process. Areas for improvement of the process were identified. Strategies to improve response rates should be explored.

## Introduction

Evidence-informed clinical practice guidelines are essential tools to support high-quality decision-making in healthcare [1, 2]. Ideally, they translate a combination of scientific evidence, clinical experience, values, and preferences into clinical practice recommendations that are relevant to the diagnosis and treatment of individual patients [3]. Their validity relies on a transparent development process using internationally accepted standards and methods [2–4]. The methods employed should be documented and made accessible and can be assessed using validated tools such as AGREE-II [5].

The trustworthiness of clinical practice guidelines also depends on implicit factors that are not typically recorded in guideline documents. This includes the adequacy of time allotted to each task, panel member selection or conduct, or the adequacy of conflict-of-interest management. Such implicit factors may be evaluated using the PANELVIEW instrument, which was created to evaluate the process and outcome of clinical practice guideline development from the perspective of the guideline group [6]. The instrument itself was developed with a focus on transparency and stakeholder involvement and then tested with eight international guideline groups. It consists of 15 domains with a total of 34 items (see Table 1).

**Table 1:** Characteristics of study participants

	Domain	CC1 (*N* = 13)	CC2 (*N* = 12)	CC3-5 (*N* = 20)
Primary role^b^:	Clinical expert (*N*)	10	10	15
	Patient representative (*N*)	0	0	0
	Policymaker (*N*)	0	0	2
	Panel chair/co-chair (*N*)	1	1	2
	Methodologist (*N*)	2	1	1
	Other (steering group member) (*N*)	1	1	1
Previous participation in guideline development:	*N* participants in previous guidelines	8	9	14
	*N* previous guidelines (mean^a^)	4	3	3
Previous roles^b^:	Panel member (*N*)	7	8	17
	Methodologist (*N*)	2	0	1
	Panel chair (*N*)	1	1	3
	Other (writing group/steering group/consulting) (*N*)	1	2	1
Formal education:	Medicine	10	8	15
	Nursing	0	2	1
	Epidemiology	1	0	0
	Natural sciences	1	1	1
	Other (‘Master’; ‘BSc’)	1	0	1
Formal training in health research methodology/epidemiology/biostatistics:	None	0	0	2
	Some, but no graduate degree	6	6	8
	MSc degree	3	2	3
	PhD degree	4	5	6
Fields of work^b^:	Clinical/health professional	11	10	16
	Policymaking	1	0	2
	Patient advocacy	0	0	0
	Research	8	7	10
	Teaching	6	7	12
	Administrative	0	1	0
	Other (retired)	0	0	1

^a^Mean of answers provided numerically (answers such as ‘several’ were discounted); ^b^ multiple answers possible. *CC*, consensus conference(s)

The German evidence- and consensus-based guideline on the treatment of patients with severe/multiple injuries (‘German polytrauma guideline’) [7] is an interdisciplinary and interprofessional guideline initially published in 2002, with updates scheduled every 5 years [8]. The guideline contains over 300 recommendations across 39 topic areas and covers the diagnosis and management of various types of injury in the prehospital, emergency room, and primary surgical setting. It is coordinated and funded by the German Society for Trauma Surgery (DGU), a non-profit scientific medical society, and its development involves delegates representing > 20 scientific medical societies and additional organisations such as emergency services associations. Based on the results of systematic evidence reviews, teams of content experts propose the wording and strength of recommendations using a three-level scheme for grading recommendations endorsed by the Association of the Scientific Medical Societies in Germany (Arbeitsgemeinschaft der Wissenschaftlichen Medizinischen Fachgesellschaften, AWMF) [9]. Delegates of all medical societies and organisations involved (‘guideline group members’) discuss these proposals during structured consensus conferences and vote on the final consensus wording and strength following a structured consensus process with neutral moderation in line with the requirements of the AWMF guidance for guideline projects.

The objective of this study was to evaluate the 2022 update of the German polytrauma guideline from the perspective of the guideline group and to identify areas where this process may be improved in the future. This is the first published application of PANELVIEW to guideline updating following its release. During finalisation of the manuscript, an application of the instrument to guideline adoption/adaptation/development (adolopment) was published by Khabsa et al. [10].

## Methods

### Study design

This survey study is reported in accordance with the Consensus-Based Checklist for Reporting of Survey Studies (CROSS) [11].

### Data collection methods

We used the German-language translation of the PANELVIEW instrument [12]. The instrument consists of 15 domains with a total of 34 items, and agreement with each survey item is rated on a 7-point Likert scale (7: fully agree; 1: fully disagree) [6]. There is an option to select ‘not applicable’. At the end of the survey, free-text fields are provided for comments on other factors that may have influenced the appropriateness of the process and/or satisfaction of the guideline group and further comments on the guideline development process.

### Sample characteristics

All contributors to the guideline process who participated in at least one of the five consensus conferences were invited to participate: the steering committee including the guideline chair and methodologist, the interdisciplinary guideline group composed of delegates of participating societies/organisations, and an independent moderator affiliated with the AWMF, as well as clinical experts that led the writing groups for recommendations discussed during the consensus conferences. Eligible persons were encouraged to participate in the survey following each of the consensus conferences they participated in.

### Survey administration

The survey was set up by the PANELVIEW team at McMaster University in Canada and conducted online via SurveyMonkey (SurveyMonkey, Momentive Inc, San Mateo, California, USA, www.momentive.ai). Consensus conferences took place on 14 June 2021, 13 September 2021, 26 January, 14 February, and 15 March 2022. Three separate survey links were generated after the first, second, and final consensus conference. The third survey was administered to all participants of the three consensus conferences that took place in 2022. Within up to 7 weeks following completion of the first, second, and set of three final consensus conferences, the guideline office sent a survey link to all participants. A reminder was sent after 2 to 4 weeks, and the survey was closed after 4 to 8 weeks. Responses were voluntary and anonymous.

### Ethical considerations

Administration of the PANELVIEW instrument to guideline groups is covered by ethics approval (Hamilton Integrated Research Ethics Board Project #14–867). Publication of the aggregate results was approved by the guideline-developing organization (German Society for Trauma Surgery (DGU)). No additional ethics approval was required because this survey was a process evaluation, the subjects were experts surveyed on a subject deemed to be within their professional competence, and the survey was voluntary and anonymous. Potential participants were informed upfront that by completing the survey, they gave their agreement that the data collected would be used for the study and would be summarized in aggregate in a publication.

### Statistical analysis

Likert scores were analysed by computing the mean with its standard deviation for each item using Excel (Microsoft Excel for Microsoft 365 MSO, Version 2208). No data were imputed. All comments obtained from the free-text fields were documented and used for internal quality improvement purposes.

## Results

Fifty-seven persons participated in at least one consensus conference and received an invitation to the survey. Among them, 21 (36.8%) received invitations for one, 9 (15.8%) for two, and 27 (47%) for all three survey rounds, so there was substantial overlap in potential survey respondents. Response rates were 36% (*n*/*N* = 13/36) for the first consensus conference, 40% (12/30) for the second, and 37% (20/54) for the set of three final consensus conferences. The number of participants (*N*) depended on the number of topics discussed in each consensus conference, because those topic experts who were not delegates of participating societies participated only in some consensus conferences. *N* is higher especially for the third survey round, because this included three separate consensus conferences with multiple topics.

Characteristics of participants who responded to the survey are detailed in Table 1. Respondents were mainly clinical experts. Other roles included the panel chair/co-chair, methodologists, and steering group members. This reflects the composition of the guideline group. No patient representatives agreed to participate in the development of this guideline, and therefore, none were available to participate in the survey. Around three quarters of participants had previous experience in guideline development, most often as panel members. The majority had a formal education in medicine, others in nursing, epidemiology, or natural sciences. Formal training in research methodology ranged from some training to a PhD degree. Participants were mainly clinical/health professionals; many were also active in research and/or teaching.

The mean scores for items ranged from 5.1 to 6.9 on a scale from 1 (fully disagree) to 7 (fully agree), as shown in Table 2. Items with mean scores below 6.0 were related to (a) four items in the domain ‘administration’, (b) consideration of patients’ views, perspectives, values, and preferences, and (c) the discussion of research gaps and needs for future research.

**Table 2:** Responses collected by the guideline group during consensus conferences 1, 2, and 3–5

		CC1	CC2	CC3–5
	Response rates [*n*/*N*, %]	13/36, 36%	12/30, 40%	20/54, 37%
Domain	Item	Mean (SD), n.a. [*n*]	Mean (SD), n.a. [*n*]	Mean (SD), n.a. [*n*]
1. Administration	1—The logistical support provided for organization of the guideline project and panel meeting was appropriate (e.g., scheduling of meeting, sharing of materials, venue/location)	5.62 (1.21) 0	6.75 (0.43) 0	5.75 (1.84) 0
	2—There was adequate preparatory work and meetings/teleconferences before the final panel meeting	5.31 (1.43) 0	6.33 (0.75) 0	5.35 (1.82) 0
	3—Adequate time was given for guideline group members to complete tasks (e.g., surveys, providing feedback) throughout the development of the guideline and to review the evidence summary and other material before the panel meeting	5.08 (1.73) 0	6.25 (0.83) 0	5.60 (1.69) 0
	4—Adequate time was allotted for the final panel meeting for all guideline questions to be discussed and recommendations to be formulated	5.92 (0.83) 0	6.08 (1.44) 0	5.68 (1.69) 1
	5—The panel meeting had a clearly defined agenda and objectives	6.82 (0.39) 2	6.67 (0.47) 0	6.84 (0.49) 1
2. Training	6—Information was provided about the specific methodology and frameworks to ensure understanding of the overall process and steps that would be used to develop the guideline	6.62 (0.62) 0	6.25 (1.16) 0	6.15 (1.56) 0
3. Panel chair	7—The panel chair(s) was able to provide clinical and methodological guidance during the meeting, providing direction and support for decision-making	6.42 (0.86) 1	6.64 (0.48) 1	6.58 (1.18) 1
	8—The panel chair(s) was able to manage the group process, establishing an atmosphere of support that ensured involvement of all panel members in the discussion and free expression of opinions	6.08 (0.95) 1	6.75 (0.43) 0	6.79 (0.69) 1
4. Conflict of interest	9—There was appropriate management of potential interests (financial, academic) of guideline group members, of the organization and in the evidence synthesis being free from bias	6.75 (0.43) 1	6.75 (0.43) 0	6.11 (1.29) 1
	10—There was appropriate management of potential bias in panel members’ interpretation of evidence and alignment with prior beliefs	6.58 (0.49) 1	6.58 (0.49) 0	6.28 (1.24) 2
5. Scoping the guideline	11—The panel was given sufficient opportunity to be involved in the prioritization of questions and scoping of the guideline	6.58 (0.49) 1	6.27 (1.14) 1	6.39 (0.83) 2
	12—The final scope of the guideline was clearly communicated to the guideline group and agreement was sought	6.55 (0.66) 2	6.45 (0.78) 1	6.61 (0.68) 2
6. Methodology and process	13—The evidence synthesis was rigorous	6.45 (0.66) 2	6.55 (0.66) 1	6.15 (1.28) 0
	14—A transparent and usable summary of the evidence was made available for the discussion	6.45 (0.66) 2	6.55 (0.66) 1	6.32 (1.17) 1
7. Considering the evidence and contributing through expertise	15—Appropriate consideration was given to the evidence, including all relevant types, and balanced with panel members’ input and opportunity to use their experience to interpret the evidence	6.25 (0.83) 1	6.82 (0.39) 1	6.25 (1.48) 0
	16—The method or process used for decision-making with the available evidence was appropriate	6.67 (0.47) 1	6.64 (0.64) 1	6.21 (1.44) 1
	17—There was appropriate involvement and consultation with key stakeholders during the guideline development	6.40 (0.66) 3	6.00 (0.82) 3	6.32 (1.03) 1
	18—Appropriate consideration was given to patients’ views, perspectives, values and preferences	5.90 (1.22) 3	5.50 (1.58) 4	5.53 (1.89) 5
8. Formulating the recommendations	19—An appropriate method was used for formulating the recommendations with transparency of judgments made	6.50 (0.50) 1	6.64 (0.64) 1	6.68 (0.57) 1
	20—Appropriate consideration was given to relevant external factors (e.g., policy implications, setting-specific health care factors, acceptability of recommendations) in formulating the guideline recommendations	6.45 (0.66) 2	6.50 (0.50) 0	6.63 (0.58) 1
	21—The consensus method used by the panel was appropriate, allowing ability to reach consensus	6.42 (0.49) 1	6.73 (0.45) 1	6.68 (1.13) 1
	22—The wording of the guideline recommendations formulated was clear and actionable	6.55 (0.50) 2	6.83 (0.37) 0	6.74 (0.64) 1
	23—There was transparency in going from the panel’s recommendations to the final recommendations that appear in the guideline report, and notice was given about any changes made	6.50 (0.50) 1	6.90 (0.30) 2	6.65 (0.65) 0
9. Group composition	24—There was diversity in membership and adequate representation of backgrounds, specialties, and balance of expertise in the panel composition	6.75 (0.43) 1	6.67 (0.47) 0	6.68 (0.65) 1
	25—The panel size was appropriate	6.58 (0.49) 1	6.73 (0.45) 1	6.21 (1.47) 1
10. Group roles	26—The required commitment was at an appropriate level for the guideline group members	6.33 (0.85) 1	6.58 (0.49) 0	6.32 (0.92) 1
	27—The contributions of the guideline group members were valued and appropriate credit was given	6.25 (0.72) 1	6.50 (0.67) 2	6.53 (0.88) 1
11. Group interaction	28—There was mutual respect between guideline group members, with friendly and professional conduct	6.42 (0.64) 1	6.70 (0.46) 2	6.68 (0.65) 1
12. Implementation and dissemination planning	29—Appropriate consideration was given to the discussion of research gaps and needs for future research	6.00 (1.04) 0	6.27 (0.75) 1	5.90 (1.41) 0
	30—Appropriate consideration was given for the planning of dissemination and implementation of the guideline	6.08 (0.86) 1	6.13 (0.78) 4	6.24 (1.44) 3
13. Writing guideline	31—The writing of the guideline was well planned, with agreement on the format(s) and opportunity for panel members to provide input and review the guideline draft	6.20 (0.60) 3	6.30 (1.00) 2	6.53 (0.78) 3
14. Incentive	32—I felt that my involvement in the guideline will have an impact on the health of people	6.08 (1.19) 1	6.73 (0.45) 1	6.56 (0.60) 2
15. Overall satisfaction	33—Overall, I was satisfied with the guideline development process	6.54 (0.50) 0	6.60 (0.66) 2	6.30 (1.35) 0
	34—I would participate in this guideline development process again	6.83 (0.37) 1	6.90 (0.30) 2	6.63 (1.13) 1

*CC* consensus conference(s), *n.a*. not applicable

## Discussion

The responses collected by the guideline group after each consensus conference were very positive overall, with a mean score for most items between 6 and 7. Between the three surveys, no major differences or trends over time were observable. The good results may be associated with the experience of the participants, around three quarters of whom had prior experience with guideline development, several within the same guideline group. In addition, this was a guideline update and largely followed previously established processes.

Items with slightly lower mean scores between 5 and 6 were related to administration, consideration of patients’ perspectives, and the discussion of research gaps. The lower mean scores in the field of administration may in part be associated with the high workload for guideline group members in this guideline: There were five all-day online consensus conferences, during which more than 300 recommendations were discussed, along with all required preparation and follow-up tasks. Other possible reasons may include technical issues with the content management system and with communication processes. Also due to the high workload, the discussion was highly focused on the evidence and recommendations, and there was little time left to address research gaps. The direct consideration of patient views was not possible because the guideline group did not include a patient representative. Patient involvement in this guideline update was planned, but we did not succeed in recruiting patients. A reason for this is the absence of patient organisations or self-help groups in the area of major trauma in Germany.

The response rate to the PANELVIEW survey was in the range of 35 to 40%. There are currently no documented figures to compare this with, though the developers of PANELVIEW observed response rates of 50–90% (personal communication). Our response rate appears adequate in the context of the substantial workload for guideline group members and the voluntary nature of the survey.

PANELVIEW has previously been used in a pilot test of eight guideline panels consisting of 94 panellists reported by Wiercioch et al. [6]. In these panels, described as ‘high performing’, mean scores for items ranged from 5.5 to 6.8. These values are similar to those we observed. Several items with mean scores below 6.0 overlap between our results and those published by Wiercioch et al., i.e. those related to administration and to the consideration of patients’ perspectives. Mean scores were higher in our project for management of potential bias in panel members’ interpretation of evidence (item 9), involvement and consultation with key stakeholders (item 17), and writing of the guideline (item 31). Khabsa et al. recently reported an average of 6.47 (SD = 0.18) across all items for their guideline adolopment process for the treatment of rheumatoid arthritis (RA) in Saudi Arabia [10]. This is also comparable to our result, although we did not calculate an average across all items. Similar to our study, the RA guideline was also supported by a very experienced guideline development group.

The study has some limitations. First, the instrument and its German-language translation has been developed using rigorous methods, but is not validated yet. Second, the threshold (mean scores below 6.0) we used to identify areas for quality improvement of the guideline was chosen arbitrarily. With a lower value e.g. of 5.0, all items would have been above the threshold. Finally, the surveys were started between 3 days and 7 weeks after the consensus conferences. However, it appears that this variable time lag has not substantially affected the response rates.

## Conclusion

The guideline development group of the German polytrauma guideline was satisfied overall with the development process, methods, and outcomes, as evaluated by the PANELVIEW instrument. Areas for future quality improvement include administration, the involvement of patients, and the discussion of needs for future research.

## Data Availability

Aggregate data is provided within the manuscript. Raw data and anonymised individual comments are available from the authors on reasonable request.
